# MediLabSecure: A decade of One Health actions to prevent emerging vector-borne diseases in the Mediterranean, Sahel and Black Sea regions

**DOI:** 10.1016/j.onehlt.2026.101393

**Published:** 2026-03-24

**Authors:** Vanessa Lagal, Elisa Pérez-Ramírez, Guillain Mikaty, Laura Amato, Claudia Robbiati, Igor Pajovic, Nebojsa Sekulic, Aleksandra Ignjatović Ćupina, Mihaela Kavran, Nabil Haddad, Rita Feghali, Sylvia Karam, Wasfi Fares, Henda Triki, Maud Seguy, Jovita Fernández-Pinero, Pilar Aguilera-Sepúlveda, Silvia Declich, Cedric Marsboom, Miguel Angel Jiménez-Clavero, Florence Fournet, Paolo Calistri, Guy Hendrickx, Maria Grazia Dente, Jean-Claude Manuguerra

**Affiliations:** a Institut Pasteur, Université Paris Cité, Department of International Affairs, Paris, France; b Centro de Investigación en Sanidad Animal (CISA-INIA), CSIC, Valdeolmos 28130, Madrid, Spain; c Institut Pasteur, Université Paris Cité, Environment and Infectious Risk Research Unit - Laboratory for Urgent Response to Biological Threats (ERI-CIBU), Paris, France; d Centro di Referenza Nazionale per l'Epidemiologia Veterinaria, la Programmazione, l'Informazione e l'Analisi del Rischio Istituto Zooprofilattico Sperimentale dell'Abruzzo e del Molise "G. Caporale", Teramo, Italy; e National Centre for Global Health, Istituto Superiore di Sanità, Rome, Italy; f Laboratory for Applied Zoology, Biotechnical Faculty, University of Montenegro, Podgorica, Montenegro; g Center for Control and prevention of infectious diseases, Institute of Public Health of Montenegro, Podgorica, Montenegro; h University of Novi Sad, Faculty of Agriculture, Center of Excellence-One Health Vectors and Climate, Novi Sad, Serbia; i Laboratory of Immunology and Vector-Borne Diseases, Faculty of Public Health, Lebanese University, Fanar, Lebanon; j Division of Health Professions, Faculty of Health sciences, American University of Beirut, Beirut, Lebanon; k Department of Laboratory Medicine, Lebanese American University Gilbert and Rose Marie Chagoury School of Medicine, Blat, Lebanon; l Department of Laboratory Medicine, Rafik Hariri University Hospital, Beirut, Lebanon; m Clinical virology laboratory, Institut Pasteur de Tunis, Tunis, Tunisia; n Research Department, Avia-GIS, Zoersel, Belgium; o Spatial Epidemiology Lab (SpELL), Université Libre de Bruxelles, Brussels, Belgium; p CIBER of Epidemiology and Public Health, Madrid, Spain; q MIVEGEC Unit, Université de Montpellier, Institut de Recherche pour le Développement (IRD), Centre National de Recherche Scientifique (CNRS), Montpellier, France

**Keywords:** Vector-borne diseases, Zoonoses, Preparedness, One Health, Regional network, Integrated surveillance, Capacity building

## Abstract

The rising incidence of vector-borne diseases driven by environmental degradation and climate change poses significant risks for human health. The implementation of integrated surveillance systems is of paramount importance to efficiently mitigate their emergence and spread.

MediLabSecure (MLS) is a pioneer One Health network launched by the European Union in 2014 to strengthen preparedness and response capacities to tackle vector-borne diseases (VBD) in the Mediterranean, Black Sea and Sahel regions, which are known hotspots for endemic and emerging vector-borne pathogens.

MLS unites experts from human health, animal health and entomology across 24 countries to foster cross-sectoral collaboration through capacity building, networking, and regional cooperation. By promoting the development and implementation of integrated surveillance systems, MLS facilitates coordinated pathogen detection, vector monitoring, risk assessment, and control strategies that encompass the human-animal-environment interface.

Over its ten-year history, the network has substantially strengthened technical capacities enabling improved arbovirus detection in human and animal samples, vector identification mapping and control, and integrated surveillance and risk assessment.

The network experience demonstrates the critical importance of coordinated multisectoral actions, regional partnerships, and long-term investments in capacity building to combat existing and future health threats at the human-animal-environment interface.

The bottom-up approach of the project, tailored to the specific needs of the beneficiary countries along with its long-term implementation – which fostered trust and stable collaborative initiatives - were key factors contributing to the success of MLS.

This unique project offers a valuable blueprint for other regions aiming to implement One Health strategies to address complex transboundary public health challenges.

## Highlights

• A unique One Health network engaged in emerging vector-borne diseases prevention in the Mediterranean Sahel and Black Sea regions.
• Enhanced capacities in virus detection, vector control and integrated surveillance.
• Strengthened One Health awareness, advocacy, and adoption in participant countries.
• A Long-term strategy that fostered trust, cross-sectoral teamwork, and sustainable impact.

## 1 Introduction

Since the late 20th century, the world has witnessed an increase in the frequency and scale of infectious disease epidemics, especially zoonotic ones, due to a complex interplay of environmental, social and biological factors [1]. Accelerating urbanization, intensified global travel, intensive agriculture and livestock farming, international trade of animals and their products, deforestation and climate change have significantly reshaped the interactions between humans, animals and ecosystems, creating more opportunities for pathogens to emerge and spread [2]. This is particularly evident for vector-borne pathogens, such as arboviruses, which are viruses transmitted by arthropods, primarily mosquitoes, ticks and sandflies. Changing conditions have expanded habitats for vectors such as *Aedes* mosquitoes and *Hyalomma* ticks [3], [4], [5] leading to the (re)emergence of vector-borne diseases (VBD) in new areas, including West Nile fever in North Africa and dengue in Europe [6], [7], [8].

Arboviruses can have a significant sanitary and socioeconomic impact due to their capacity to cause widespread outbreaks in humans and animals, livestock losses, reduced productivity, trade restrictions and public health emergencies [9]. The absence of specific therapeutics or effective vaccines for many arboviral diseases further underscores the critical role of preventive strategies, particularly coordinated surveillance plans, to enable early detection and timely outbreak mitigation measures.

Efficient management of these diseases requires a holistic approach that integrates all relevant sectors: human, animal and environmental health.

The One Health (OH) approach provides a comprehensive framework for implementing integrated surveillance and response. Developed in the early 21st century, the OH concept has evolved into an integrated and unifying approach aimed at sustainably balancing and optimizing the health of humans, animals, plants and ecosystems, acknowledging their interconnectedness [10]. The OH concept is particularly well-suited to VBD as their control requires an integrated approach considering vectors and their environment, humans, and, in the case of zoonoses, animals.

Recognizing the growing sanitary and economic impact of VBD the European Union, through its CBRN Centres of Excellence initiative, funded the MediLabSecure (MLS) project in 2014 to address these emerging health threats in the Mediterranean, Sahel and Black Sea regions. The global aim of the project was to support countries to tackle vector-borne diseases by adopting a OH approach by strengthening preparedness and responses capacities of a multidisciplinary institutional network. The project served as a platform for OH awareness and multisectoral collaboration towards integrated surveillance.

This article reviews the key actions and achievements from the 10-year implementation of MediLabSecure, highlighting lessons learned in establishing a cohesive and long-lasting regional OH network. The article also explores future directions for building on the project's legacy and further strengthen this unique OH network.

## 2 Project strategy

**Fig. 1:**
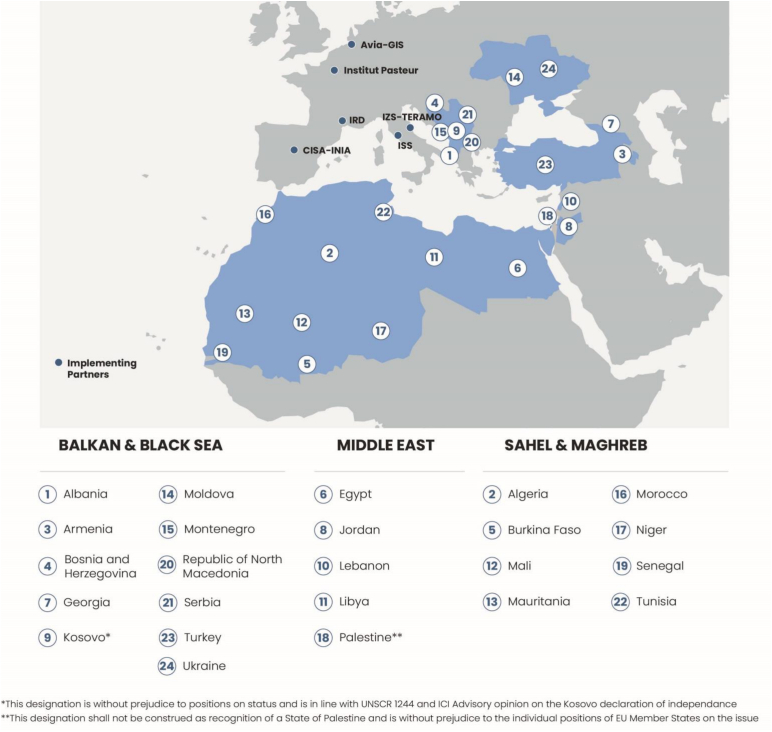
The MediLabSecure network unites over 400 multidisciplinary experts (virologists, entomologists, epidemiologists, veterinarians, and modelers) from more than 120 national reference laboratories and public and animal health institutions in up to 24 EU neighbouring countries of the Balkan, Black Sea, Middle East, Maghreb and Sahel regions.

MediLabSecure project was implemented from 2014 to 2024 and targeted up to 24 EU-neighbouring countries across the Mediterranean, Black Sea and Sahel regions (Fig. 1). This extended geographical area was identified as high-risk for VBD outbreaks, laying at the crossroads of three continents and characterized by intense trade, tourism and human migrations. It includes diverse yet connected ecosystems, with the targeted countries sharing common disease priorities and similar vulnerabilities to global change.

The project adopted a strategy based on the establishment of a regional network of national reference laboratories and public and animal health institutions. In alignment with the OH approach, the initiative brought together multidisciplinary experts from five complementary sectors involved in VBD management: human and animal virology, medical entomology, public health and veterinary services. The project was jointly implemented by a consortium of six European Union partner institutions, each representing one of the core sectors mentioned above (Fig. 1).

MediLabSecure adopted a progressive and comprehensive capacity-building approach tailored to the specific needs expressed by network members through regular surveys. It first focused on strengthening technical competencies within individual sectors to ensure further efficient cross-sectoral collaboration. To promote collaboration across sectors, the project organized networking events to raise awareness on the OH concept and to foster connections among experts from different disciplines at the national and regional levels. Building on these connections, MediLabSecure subsequently implemented joint activities and collaborative practical exercises designed to encourage multidisciplinary teamwork.

Based on the priority VBD identified in the area [11], MLS centered its capacity building program on three emerging pathogens: West Nile virus (WNV), Crimean-Congo haemorrhagic fever virus (CCHFV) and Rift Valley fever virus (RVFV) with additional actions on dengue, chikungunya, tick-borne encephalitis and zika viruses [12].

## 3 Outcomes

### 3.1 Preparedness and response

Effective national response to emerging VBD requires not only robust laboratory detection capabilities, but also effective surveillance systems for animals and vectors since a proactive approach is far more effective and less costly than responding to an outbreak [13]. Early detection of viral circulation in reservoir hosts and vectors enables timely control measures—such as vector control and animal vaccination—that considerably reduce disease impact [14]. Strengthening diagnostic capacities in human and animal virology labs, along with improving vector identification skills, is essential to enhance surveillance and control efforts across sectors [15]. The needs assessment conducted at the beginning of the project revealed that 63 and 79% of the human virology laboratories were capable of detecting dengue and WNV, respectively. In contrast, detection capacities for other arboviruses were significantly lower, ranging from 5% to 17%. For animal virology laboratories, only 37.5% had well established protocols to diagnose RVFV, and 56% to detect WNV [14], [16]. Moreover, several participating countries reported gaps in vector identification skills.

To address the identified capacity building needs, the project developed an ambitious training program that included workshops, production and sharing of standard operating procedures, provision of positive controls and diagnostic reagents, and the development of sustainable tools for vector identification. In total, 30 training workshops, including hands-on laboratory sessions, were organized covering (1) molecular and serological diagnostic techniques for arboviruses including biosafety and biosecurity practices, (2) next-generation sequencing and metagenomics and (3) field diagnostic in mobile laboratories. To improve entomological capacities, 16 training sessions were organized on mosquito identification, vector mapping and monitoring of insecticide resistance. Moreover, five vector identification tools were produced including electronic free and interactive tools to identify mosquitoes and phlebotomines of the Euro-Mediterranean region (access tools at references [17], [18]).

A notable strength of the project was the implementation of post-training evaluations, including External Quality Assessement (EQA) exercises to ensure effective application of gained knowledge. The assessments evaluated VBD diagnostic performance in human and animal samples [14], [16], [19] and on mosquito identification skills using direct identification of adults and larvae [20] and high-definition photomicrographs [21]. Results showed significant improvements, with 85–100% of virology laboratories accurately identifying targeted arboviruses [14], [16], [19] and entomological species identification accuracy increasing from 64% to 82% [20], [21].

The positive impact of the capacity building activities is illustrated by two successful examples.

The human virology laboratory in Institut Pasteur de Tunis improved its technical skills for WNV surveillance including molecular and serological diagnosis, sequencing and genomic data analysis. These achievements strengthened the national surveillance system, which progressively integrated WNV in response to recent recurrent epidemics in the country. Building on these advances, the institution organized a regional training on field outbreak investigation with multidisciplinary participants from Tunisia, Algeria and Libya – countries facing similar environmental and epidemiological risks. These collaborative efforts led to the establishment of cross-border and multisectoral surveillance, exemplifying operational implementation of OH systems at the regional level.

A second success story was the pilot study led by the animal virology coordination team at INIA-CSIC, in collaboration with five veterinary laboratories from the Balkans and Black Sea regions for serological diagnosis of CCHFV. The study developed a two-step method with an initial ELISA screening followed by immunofluorescence confirmation, combining high sensitivity in the screening phase with high specificity in the confirmation phase [22]. This approach confirmed CCHFV exposure in animals in Montenegro and Bosnia and Herzegovina for the first time [8] and provided the first evidence of viral exposure in animals in Georgia.

### 3.2 Technical support during sanitary emergencies

Over its ten years, the project provided 28 technical assistance and diagnostic resources to MLS beneficiary countries facing outbreaks and epidemics. These actions contributed to improving early response and the establishment of control measures.

Although COVID-19 is not a VBD, its exceptional impact prompted rapid mobilization of most of the human virology laboratories in beneficiary countries as frontline diagnostic laboratories. In some countries, animal virology laboratories were also involved in addressing the overwhelming diagnostic demand. MLS played a key role in facilitating the rapid dissemination of diagnostic protocols, positive controls and essential reagents. The project also supported the sequencing of the initial strains and emerging variants, while providing targeted training to the laboratories involved in genomic surveillance and virus characterization [23], [24]. Moreover, the project organized training sessions on SARS-CoV-2 sequencing. Considering the zoonotic character of the disease, an online webinar about diagnosis of SARS-CoV-2 in animals was organized to respond to the need of the veterinary laboratories that were receiving samples from pets, livestock and wildlife. In addition, training materials compiling information about susceptibility, clinical signs and epidemiology of the disease in different animal species were produced with several updates during 2020 and 2021 (access document at reference [25]).

In-depth description of the actions provided by MLS during the pandemic is available in Mikaty et al., 2026 [26].

Laboratories within the network acknowledged that the fast provision of reagents was instrumental in establishing diagnostic techniques and, in several cases, enabled the identification of their first COVID-19 cases.

Additionally, the project expanded support to frontline healthcare workers by developing an offline training program to strengthen COVID-19 surveillance and protection in low-income countries. It also supported a multicenter surveillance study in five African countries to assess healthcare workers' infection risk and protective practices [27], [28], [29], [30], [31], [32].

### 3.3 One Health awareness

MediLabSecure engaged the country stakeholders from different sectors in a long-term raising awareness process to promote the One Health (OH) approach for addressing VBD and building necessary capacities and resources.

Six MediLabSecure Situational Analysis (MeSA study) were carried out in pilot countries (Georgia, Tunisia, Serbia, Montenegro, Armenia, and Bosnia and Herzegovina) [33], [34], [35], [36], [37]. The 6 MeSA studies were in-country operational research studies engaging all the OH national actors, to document how arbovirus surveillance was integrated across sectors and to identify enabling factors to reinforce integrated surveillance.

These studies were also an opportunity to foster long lasting connections and build awareness about the importance of the OH approach for the integrated surveillance of priority pathogens. For example, in Serbia, the study contributed to consolidate the OH landscape in the country, particularly in the areas of OH governance, plans, surveillance and training, leading to the first integrated WNV national surveillance program [38], [39]. A relevant achievement was the establishment of the Centre of Excellence for One Health at the University of Novi Sad accredited by the Ministry of Science, Technological Development and Innovation of the Republic of Serbia in 2021. The Centre brings together experts across disciplines from different Serbian institutes (the Faculty of Medicine, the Institute of Public Health of Vojvodina and the Veterinary Institutes). This progress has been driven by strong informal interdisciplinary collaborations that eventually became formalized, underpinned by a clear willingness to work across sectors (access website at reference [40]).

Additionally, OH awareness was promoted through regional meetings where network members and stakeholders from different sectors gathered and reflected on challenges to OH implementation, to establish intersectoral collaborations and to foster relevant initiatives for better VBD surveillance in the countries [41].

Finally, OH awareness was also achieved through the organization of six multisectoral exercises covering various topics in different formats: tabletop exercises on outbreak investigation, setting up of integrated surveillance, multisectoral transfer knowledge or serious game such as ALERT (funded by the EU and developed by WOAH, CIRAD, IRD, and Institut Pasteur in collaboration with Bioviva). The main objectives of these exercises were to advocate the importance of sharing information between sectors and to inform the participants about the correct flow of information during the investigation and management of zoonotic disease outbreaks. By bringing together experts from different sectors (virologists, epidemiologists, entomologists, public health experts, etc.) the exercises fostered collaboration within and between countries.

Another illustration of the project's successful multisectoral networking approach is the fostering of collaboration between experts from human virology, animal virology, entomology and epidemiology in Montenegro. Together, they developed the 2023–2025 National Vector Surveillance and Control Plan, which has been endorsed by the government to promote a rapid response against vector-borne sanitary threats affecting public health (access document at reference [42]).

### 3.4 Integrated surveillance

The practical application of the OH approach requires the implementation of integrated and collaborative actions to strengthen surveillance, risk assessment and early warning systems for VBD. While capacity-building and training activities are essential, their long-term impact may be limited unless they are accompanied by practical measures that enable cross-sectoral coordination in day-to-day operations.

To understand the baseline of the OH operationalization across the various beneficiary countries, multiple reviews, assessments and analyses were conducted throughout the project. These actions revealed a highly diverse landscape with relevant variations across countries and regions [11], [43].

In the framework of MLS, three multisectoral risk assessment (MRA) exercises were conducted [44]. These exercises emphasized the importance of data sharing and coordination to reduce surveillance gaps. MRAs carried out at the regional level using the OH approach have proved to be valuable tools for identifying critical vulnerabilities and informing both national and regional health security strategies.

The outcomes of the 6 MeSA studies reported in the previous section enabled identifying operational and communication gaps, and proposed ways to enhance cross-sectoral data sharing which were compiled into two Strategic Documents that provide actionable recommendations for operationalizing the OH approach within national surveillance systems (access documents at reference [45], [46]).

MediLabSecure also supported capacity building on risk mapping and GIS capacities. Training on risk mapping methods was provided during regional meetings and tailored eight-week hands-on GIS basic and medium level courses delivered through a customized distance learning platform. With these tools, participants learned to integrate multiple data layers to produce risk maps aimed at supporting decision-makers in identifying and addressing VBD threats. In addition, selected participants who successfully completed these courses received personalized support on advanced GIS topics relevant to their national context.

Altogether, these activities beyond improving technical capacities also contributed to raise awareness of the added value of cross-sectoral data integration for early warning and response to VBD. They further helped national institutions in identifying the strengths and weaknesses of their OH systems, ultimately guiding the development of context-specific implementation roadmaps.

Lebanon illustrates both the project's concrete result and its ongoing challenges. Before 2015, no WNV surveillance program existed, and monitoring was limited. A 2015–2016 multisectoral survey revealed WNV circulation in humans and horses despite no reported clinical cases [47]. MLS strengthened Lebanon's diagnostic and entomological capacities through targeted training and tools like MosKeyTool, improving vector identification. Building on these improvements, an active WNV surveillance plan in humans was launched in the country integrating WNV screening into the national meningitis surveillance program, enabling detection of potential WNV neurological cases. However, a fully integrated system has yet to be established to coordinate disease surveillance in human, animal and entomological sectors. While some of these activities are being carried out through isolated monitoring studies, only the integration of diverse data sources can enable the development of an effective early warning-based system to address this and other VBD in the country.

### 3.5 Conclusion and lessons learnt

The outcomes of the MLS project highlight the critical value of an integrated and collaborative approach to build a cohesive cross-sectoral network to address emerging biological threats. Two Strategic Documents that provide actionable recommendations for operationalizing the OH approach within countries and support integrated surveillance systems were developed [[45], [46]].

**Fig. 2:**
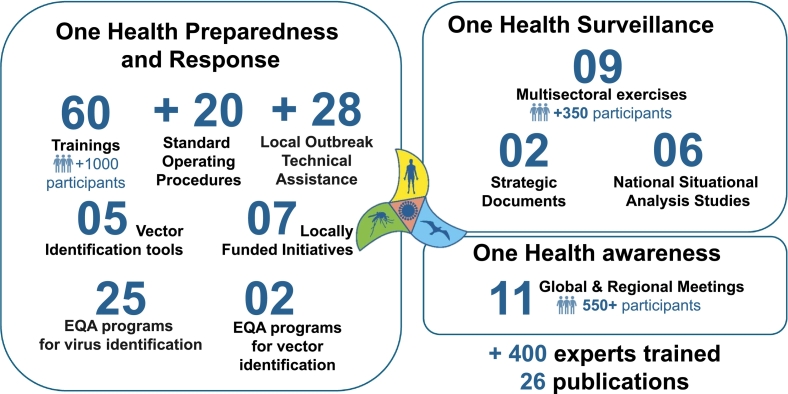
Key numbers from the 10-year actions dedicated to strengthen VBD prevention capacities in the Mediterranean region.

The key numbers of the MLS project are presented in Fig. 2.

Several key factors contributed to successfully building a regional OH network:

(1) As VBD do not respect national borders, the regional approach, which allows to address shared needs, proved to be instrumental. MLS facilitated the harmonization of preparedness and response capacities across countries, supporting coordinated cross-border surveillance that is essential for early detection and effective containment of regional outbreaks.

(2) The project curriculum followed a bottom-up approach tailored to the specific needs of participating institutions across multiple disciplines and engaged a wide range of health actors involved in VBD management. It operated on both technical and institutional levels: technically, by building capacity through training activities aimed at strengthening diagnostic and vector control competencies; institutionally, by promoting the added value of the OH approach, fostering multisectoral collaboration and supporting the establishment of integrated surveillance systems.

(3) The stepwise methodology was essential in achieving trans-sectoral cooperation. MLS first strengthened the capacities of each sector and then progressed to cross-sectoral activities, including multisectoral workshops where experts from different sanitary fields were trained in a topic of common interest, such as full genome sequencing, metagenomics analysis or transfer knowledge. The networking events and the multi-sectoral practical exercises significantly facilitated cross-sectoral cooperation at the national and regional levels.

(4) The 10-year project's continuity, made possible by the long-term EU investment, was crucial for establishing trust, creating a favorable environment for sustainable collaboration and ownership of the OH concept at least at the operational level. Over the years, experts formed multisectoral teams with different objectives such as conducting seroprevalence studies to gather evidence on VBD circulation for decision-makers, managing local outbreaks or contributing to the development of national surveillance plans.

(5) The network proved to be extremely useful in facing the COVID-19 pandemic in all beneficiary countries. Its flexible and methodological rather than disease-driven approach provided surveillance and control tools applicable to a broad range of emerging zoonotic threats as demonstrated during the pandemic. This experience highlighted the value of a strong regional network for rapid response, laboratory resilience, and access to essential resources during a global crisis. Moreover, the involvement of animal virology laboratories in COVID-19 diagnostics demonstrated the effectiveness of cross-sectoral collaboration for epidemic management.

(6) The sustainability of the network was achieved through the empowerment of network's members by funding initiatives in their countries encouraging the “train of trainers” approach and the exchange of knowledge between experts at the national or regional level [41].

(7) The flexibility of the project was key to overcoming problems faced. For instance, the project adapted to changing political, social and eco-biological contexts, minimizing deviations from the initial objectives. Needs were regularly assessed to ensure the development of tailored training programs addressing countries' health concerns. The project was able to provide support on diseases outside the scope of the project. VBD are used as a proxy for better epidemic preparedness.

The MLS model emphasizes the importance of multisectoral collaboration, local empowerment and adaptive strategic planning and it offers a promising blueprint for addressing complex, cross-border health challenges in other regions or contexts.

As global health landscapes evolve, such integrated approaches will become increasingly essential in mitigating emerging infectious disease risks and maintaining health security.

After 10 years of implementation, this unique OH network is now ready for further development. While existing gaps in VBD control can be addressed, future directions should focus on strengthening MLS holistic approach by improving collaboration with the environment sector and promoting community engagement in outbreak management. To maximize the impact of MLS, future efforts should go beyond training by supporting participating institutions in the practical implementation of OH principles in their respective countries. Taking a step further towards comprehensive OH implementation also requires investment to build the next generation of OH professionals and to foster dialogue with policymakers and help build political commitment towards the adoption of the OH approach.

## CRediT authorship contribution statement

**Vanessa Lagal:** Writing – original draft, Validation, Supervision, Project administration, Funding acquisition, Formal analysis, Data curation, Conceptualization. **Elisa Pérez-Ramírez:** Writing – original draft, Validation, Methodology, Investigation, Funding acquisition, Formal analysis, Conceptualization. **Guillain Mikaty:** Writing – original draft, Validation, Methodology, Investigation, Funding acquisition, Formal analysis, Conceptualization. **Laura Amato:** Writing – original draft, Validation, Methodology, Investigation, Funding acquisition, Formal analysis, Conceptualization. **Claudia Robbiati:** Writing – original draft, Validation, Methodology, Investigation, Funding acquisition, Formal analysis, Conceptualization. **Igor Pajovic:** Writing – review & editing, Methodology, Investigation. **Nebojsa Sekulic:** Writing – review & editing, Methodology, Investigation. **Aleksandra Ignjatović Ćupina:** Writing – review & editing, Methodology, Investigation. **Mihaela Kavran:** Writing – review & editing, Methodology, Investigation. **Nabil Haddad:** Writing – review & editing, Methodology, Investigation. **Rita Feghali:** Writing – review & editing, Methodology, Investigation. **Sylvia Karam:** Writing – review & editing, Methodology, Investigation. **Wasfi Fares:** Writing – review & editing, Methodology, Investigation. **Henda Triki:** Writing – review & editing, Methodology, Investigation. **Maud Seguy:** Writing – review & editing, Validation, Supervision, Project administration, Funding acquisition, Formal analysis, Data curation, Conceptualization. **Jovita Fernández-Pinero:** Writing – original draft, Validation, Methodology, Investigation, Funding acquisition, Formal analysis, Conceptualization. **Pilar Aguilera-Sepúlveda:** Writing – original draft, Validation, Methodology, Investigation, Funding acquisition, Formal analysis, Conceptualization. **Silvia Declich:** Writing – review & editing, Validation, Supervision, Methodology, Funding acquisition, Formal analysis, Conceptualization. **Cedric Marsboom:** Writing – original draft, Validation, Methodology, Investigation, Funding acquisition, Formal analysis, Conceptualization. **Miguel Angel Jiménez-Clavero:** Writing – review & editing, Validation, Supervision, Methodology, Funding acquisition, Formal analysis, Conceptualization. **Florence Fournet:** Writing – review & editing, Validation, Supervision, Methodology, Funding acquisition, Formal analysis, Conceptualization. **Paolo Calistri:** Writing – review & editing, Validation, Supervision, Methodology, Funding acquisition, Formal analysis, Conceptualization. **Guy Hendrickx:** Writing – review & editing, Validation, Supervision, Methodology, Funding acquisition, Formal analysis, Conceptualization. **Maria Grazia Dente:** Writing – review & editing, Validation, Supervision, Methodology, Funding acquisition, Formal analysis, Conceptualization. **Jean-Claude Manuguerra:** Writing – review & editing, Validation, Supervision, Methodology, Funding acquisition, Formal analysis, Conceptualization.

## Funding

The MediLabSecure project is fully funded by the 10.13039/501100000780European Commission (IFS/2013/330961; IFS/2018/402-247; NDICI/2022/427-564). The publication's contents are the sole responsibility of the authors and do not necessarily reflect the views of the European Union.

## Declaration of competing interest

The authors declare no conflict of interest.

## Data Availability

Data will be made available on request.
